# Risk factors for relapse or persistence of bacteraemia caused by *Enterobacter* spp.: a case–control study

**DOI:** 10.1186/s13756-017-0177-0

**Published:** 2017-01-21

**Authors:** Patrick N. A. Harris, Anna M. Peri, Anita M. Pelecanos, Carly M. Hughes, David L. Paterson, John K. Ferguson

**Affiliations:** 1 University of Queensland, UQ Centre for Clinical Research, Royal Brisbane and Women’s Hospital, Building 71/918 Royal Brisbane & Women’s Hospital Campus, 4029 Herston, QLD Australia; 2 Department of Biomedical and Clinical Sciences Luigi Sacco, University of Milan, Milan, Italy; 3 QIMR Berghofer Medical Research Institute, Herston, QLD Australia; 4 Pathology North - Hunter, John Hunter Hospital, Newcastle, NSW Australia; 5 UQ Centre for Clinical Research, Royal Brisbane and Women’s Hospital, QLD, Australia & Wesley Medical Research, University of Queensland, Toowong, QLD Australia; 6 School of Biomedical Sciences and Pharmacy, University of Newcastle, Newcastle, NSW Australia; 7 School of Rural Medicine, University of New England, Armidale, NSW Australia

**Keywords:** *Enterobacter cloacae*, *Enterobacter aerogenes*, Beta-lactamase, AmpC, Bacteraemia, Outcomes, Relapse, Treatment

## Abstract

**Background:**

*Enterobacter* spp. possess chromosomal AmpC beta-lactamases that may be expressed at high levels. Previous studies have demonstrated a risk of relapsed bacteraemia following therapy with third generation cephalosporins (3GCs). What additional factors predict microbiological failure in *Enterobacter* bacteraemia is unclear. We aimed to determine factors associated with microbiological failure in *Enterobacter* bacteraemia.

**Methods:**

We retrospectively identified cases of bacteraemia caused by *Enterobacter* spp. occurring in four hospitals. Using a case–control design, we determined clinical risk factors for persistence or relapse defined as repeated positive blood cultures collected between 72 hours and up to 28 days post initial positive blood culture.

**Results:**

During the study period a total of 922 bacteraemia events caused by *Enterobacter* spp. in adults were identified. The overall risk of relapsed or persisting bacteraemia at 28 days was low (31 of 922, 3.4%), with only 2 patients experiencing emergent resistance to 3GCs. A total of 159 patients were included in the case–control study. Using multivariate logistic regression, independent predictors for relapse were a line-associated source of infection (OR 3.87; 95% CI 1.56-9.60, *p* = 0.004) and the presence of immunosuppression (OR 2.70; 95% CI 1.14-6.44, *p* = 0.02). On univariate analysis definitive therapy with a broad-spectrum beta-lactam-beta-lactamase inhibitor (BLBLI, e.g. piperacillin-tazobactam) was not associated with relapse (OR 1.83; 95% CI 0.64-5.21, *p* = 0.26) although the proportion of patients receiving a BLBLI as definitive therapy was relatively small (21/159, 13.2%).

**Conclusions:**

The risk of relapsed or persistent *Enterobacter* bacteraemia appears to be low in Australia. A line-associated source of infection and immunocompromise were significant independent predictors for relapse. Larger, preferably randomized, studies are needed to address whether BLBLIs represent an effective carbapenem-sparing option for *Enterobacter* bacteraemia.

**Electronic supplementary material:**

The online version of this article (doi:10.1186/s13756-017-0177-0) contains supplementary material, which is available to authorized users.

## Background

Bacteria belonging to the genus *Enterobacter* spp. are Gram-negative Proteobacteria of the family Enterobacteriaceae and currently comprise 22 species [1]. They represent a diverse group which are widely distributed in nature [2] and possess multiple mechanisms to allow survival in a variety of environmental niches [3]. In humans, they may cause a wide variety of clinical infections and are a common cause of bacteraemia [2], especially within adult and neonatal intensive care units (ICUs) [4, 5].

**Fig. 1:**
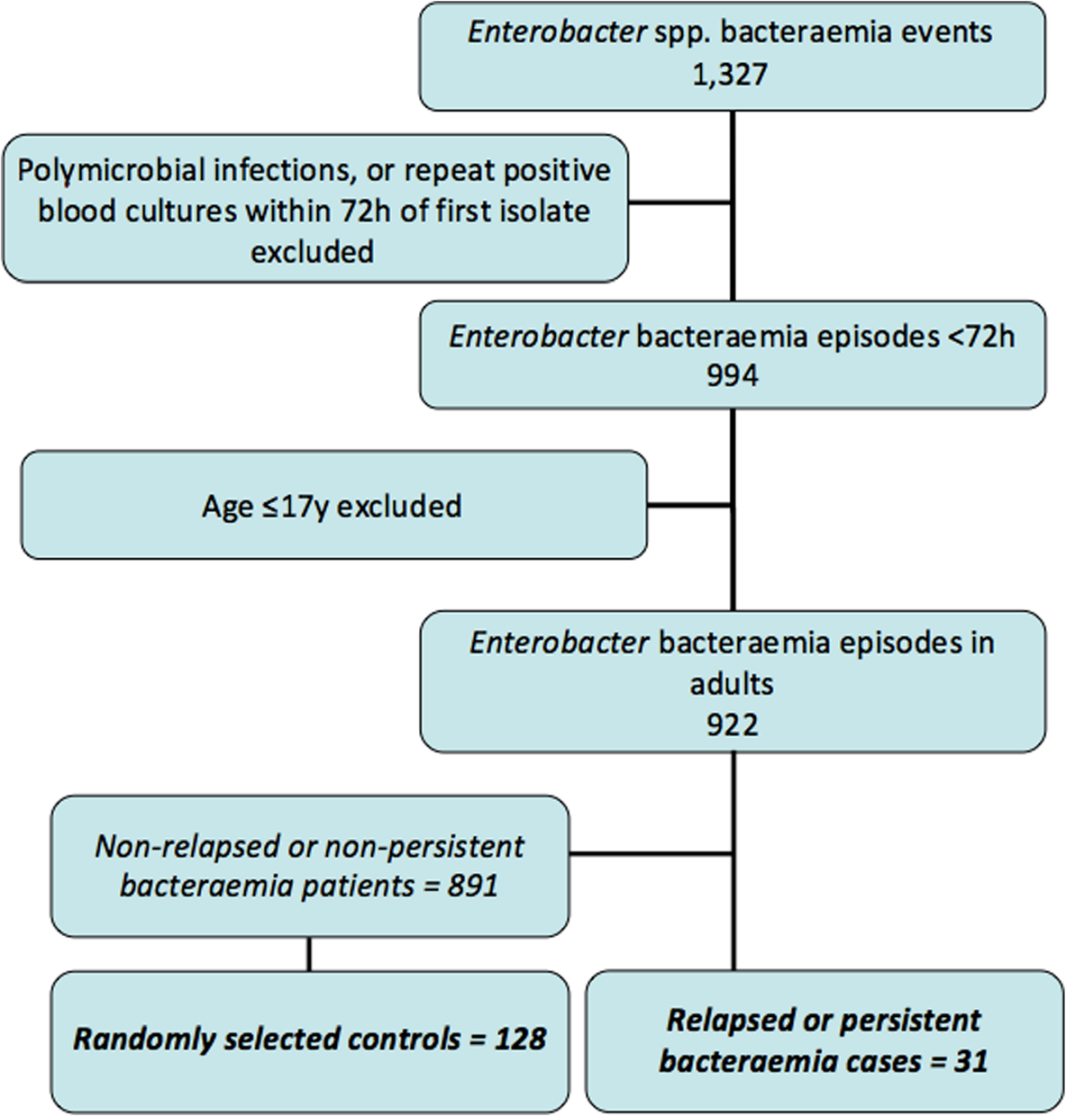
Inclusion flowchart for case–control study

The annual incidence of bacteraemia caused by *Enterobacter* spp. has been reportedly increasing in some parts of the world [6]. This bacterial genus presents particular challenges for the selection of optimal therapy due to the presence of chromosomally encoded AmpC beta-lactamase enzymes [7]. These enzymes are able to hydrolyse many beta-lactams, including third generation cephalosporins and may be induced by beta-lactam exposure. Furthermore, AmpC gene expression can become constitutively de-repressed by mutational loss of regulatory genes, leading to high-levels of AmpC production and a phenotype that demonstrates in vitro resistance to most beta-lactams and beta-lactam/beta-lactamase inhibitor (BLBLI) combination agents, except cefepime and carbapenems. [2] Such variants are usually present at low levels (e.g. between 10^−5^ to 10^−7^ of the total bacterial population) but may be rapidly selected during antibiotic therapy [2]. This phenomenon has been best described with the use of third generation cephalosporins (3GCs). In a prospective study of *Enterobacter* bacteraemia, emergence of resistance developed during treatment in 6 of 31 (19%) bacteraemia episodes treated with 3GCs [8]. Similar outcomes were replicated in a later study [9], although relapse rates have been reported as lower in other studies [10–12]. In a large cohort of patients from South Korea, emergent resistance during therapy with 3GCs was more likely to occur with a complex focus of infection (particularly malignant bile duct obstruction) but was never seen in urinary tract infections [10]. Development of resistance in *Enterobacter* infections has been associated with higher mortality and healthcare-related costs [13]. As a result, 3GCs are usually not recommended as first line therapy, even when susceptible in vitro.

*Enterobacter* may also acquire other major resistance determinants such as extended-spectrum beta-lactamase (ESBL) enzymes or carbapenemases [14, 15]. Carbapenem resistance may also develop from AmpC-hyperproduction in association with porin mutations [16] and multi-drug resistant (MDR) *Enterobacter* has been implicated in nosocomial outbreaks [17–21]. As such, *Enterobacter* spp. have become important ‘problem’ pathogens in the healthcare setting [22]. Most significant *Enterobacter* infections are treated with agents such as carbapenems, quinolones or aminoglycosides. Cefepime may be a useful option given its stability to AmpC enzymes [23]. Trimethoprim-sulphamethoxazole may also be effective [24], but is rarely used in contemporary practice for serious invasive *Enterobacter* infections. BLBLI agents, such as piperacillin-tazobactam, have an uncertain role in this context. [7] Although they are often avoided over concerns relating to the development of AmpC-mediated resistance, the risk of this occurring for this antibiotic class has rarely been examined, and may even be associated with improved outcome when used as empirical therapy in one study [25].

## Methods

The aim of the study was to determine clinical factors associated with relapsed or persistent infection in patients with *Enterobacter* bacteraemia. A specific hypothesis to be tested was that the use of BLBLI agents as definitive therapy carried no additional risk of bacteraemia relapse when compared with alternative established therapies.

### Setting

The study was undertaken across four hospitals served by two public microbiology laboratories in Queensland and New South Wales, Australia. These included the Royal Brisbane and Women’s Hospital (in Queensland), John Hunter Hospital in Newcastle, The Mater Hospital in Newcastle and Belmont Hospital (all in New South Wales).

### Study design

A case–control design was used to determine risk factors for bacteraemia relapse or persistence beyond 3 days following initial positive blood culture. Adult patients (> = 18 years of age) with laboratory confirmed *Enterobacter* spp. in at least one blood culture draw were identified from August 1998 to August 2012 from Pathology North, and between October 1999 and November 2015 from Pathology Queensland. ‘Relapsed or persistent’ cases were defined if there was any positive blood culture ≥72 hours, and up to 28 days, after the initial positive blood culture with identification of an *Enterobacter* isolate of the same species. Control patients (at a ratio of approximately 1:4) were randomly selected from those patients who had no relapsed bacteraemia during the 28 days following initial positive blood culture. For the logistic regression, control patients who died within 28 days were excluded as it was unknown whether they would have relapsed had they survived.

### Inclusion / exclusion criteria

Any patient with at least one positive monomicrobial blood culture with *Enterobacter* spp. was included. Patients aged ≤17 years at the time of infection were excluded.

### Clinical data collection

Cases were identified by data extraction from the Pathology North and Pathology Queensland laboratory information systems (AUSLAB; PJA, Melbourne). Clinical and demographic data on all significant bloodstream infections were prospectively collected as routine surveillance by the infection control and microbiology services. Relapsed cases were retrospectively identified from laboratory data. Outcomes were determined at 28 days post initial positive blood culture for all cases and controls.

In addition to demographic details, clinical variables recorded included source of infection, hospital location, co-morbid conditions, admitting clinical service, acquisition status of infection, initial antibiogram of the blood culture isolate (AmpC de-repressed phenotype), the presence of vascular access devices and the neutrophil count on the day of first positive blood culture. Data on antibiotic use and SAPS II physiology scores [26] (determined on the day of first positive blood culture) were recorded from clinical chart review. Only antimicrobial agents with Gram-negative activity were recorded, with those used within the first 48 h after initial blood culture defined as empirical use and those prescribed after blood culture results available defined as definitive use. Healthcare acquisition and source designation of the bacteraemia were categorised according to standard definitions [27–29]. Empirical antibiotic therapy was described as appropriate if the isolate was susceptible to at least one agent used. If an agent was used for ≥50% of the definitive treatment duration, this was listed as the primary agent. If a second agent was used either concurrently or sequentially, the patient was described as receiving combination therapy; if combination therapy was used for the majority of the definitive treatment duration the antibiotic choice was determined as ‘other’. For statistical analysis, if the definitive regimen included a carbapenem, quinolone, co-trimoxazole, cefepime or an aminoglycoside to which the isolate was susceptible, the treatment was classified as ‘standard therapy’, if piperacillin-tazobactam or ticarcillin-clavulanate was used and the isolate was susceptible, the treatment was classified as ‘BLBLI’, otherwise the treatment was defined as ‘inappropriate’ (e.g. cephalexin, cephazolin, ceftriaxone, ampicillin/amoxicillin, amoxicillin-clavulanate or no therapy). Standard dosing regimens at participating hospitals for piperacillin-tazobactam are 4.5 g 8-hourly and ticarcillin-clavulanate 3.1 g 6-hourly, with dose adjustment for renal dysfunction according to the Australian Therapeutic Guidelines [30]. Patients were classified as being immunosuppressed if they had the following conditions: neutropenia (neutrophil count <0.5 x10^9^/L), haematological / solid organ malignancy or myelodysplastic syndrome, solid organ transplant or if they received any other immunosuppressive drug therapy including prolonged high dose corticosteroids (≥30 mg prednisolone or equivalent daily).

### Microbiological methods

Bacterial isolates were identified by routine diagnostic methods employed in the laboratory over the study period. Bacteraemia was diagnosed using the BD BACTEC automated system (Becton Dickinson, Sydney, Australia). Species identification methods used included the Vitek 2 or API 20E systems (Biomerieux, Marcy-L’Etoile, France), BBL Crystal (Becton Dickinson), as well as MALDI-TOF (Vitek MS, Biomerieux) and routine bench testing. Susceptibility testing was performed using Kirby-Bauer disk diffusion methods or by Vitek2 automated microbroth dilution (Biomerieux), with interpretative standards as defined by the Clinical and Laboratory Standards Institute (CLSI) at the time of testing [31], although from 2012 onwards Pathology Queensland switched to EUCAST methodology [32].

### Statistical methods

Data describing patient demographics, microbiology results, clinical features, bacteraemia source, co-morbidity, acquisition status and clinical service for all patients were tabulated for relapse cases and non-relapse cases, with categorical variables expressed as percentages and median (interquartile range [IQR]), mean (standard deviation [SD]) and/or ranges calculated as appropriate for scale variables. Wilcoxon rank-sum test was used to explore differences between non-parametric continuous variables. Potential risk factors for relapse as the dependent variable were included in a univariate logistic regression model. Variables with a *p*-value of <0.2 and/or with large effect estimates (odds ratios [OR] > 2 or < 0.5) in the univariate analysis were included in the multivariate model. Odds ratios with 95% confidence intervals (CI) were calculated for predictors of relapse. The multivariate model was optimized using a stepwise approach, beginning with the univariate model demonstrating the strongest association with relapse. The goodness-of-fit of the model before and after each step was compared by the likelihood-ratio test and the optimal model fit determined using Akaike’s and Bayesian information criteria. Variables that did not significantly improve the model fit were discarded. Statistical analysis was performed using Stata 13.1 (StataCorp; TX, USA). A *p*-value <0.05 was considered significant. Using a ratio of 1:4 cases to controls, and assuming a 40% exposure to variables in the control group that may influence outcome, we calculated that we would need 35 cases and 137 controls, assuming a two-sided confidence level of 95% to achieve 80% power to detect an odds ratio of at least 3.0.

## Results

During the study periods for each location, a total of 922 positive blood cultures growing *Enterobacter* spp. were identified, after excluding polymicrobial infections, individuals aged <18 years and repeatedly positive cultures within the first 72 h after initial bacteraemia. A total of 31 (3.4%) patients were classified as having persistent or relapsed *Enterobacter* bacteraemia, occurring between 3 and 28 days post initial positive blood culture. A total of 159 patients (all 31 cases and 128 randomly-selected controls) were included in the case–control study to identify risk factors for bacteraemia relapse or persistence (see Fig. 1). The proportions of different species identified and their antimicrobial susceptibilities are shown in Table 1. The most common species identified was *Enterobacter cloacae* (76.7%). A proportion of isolates had an initial antibiogram suggesting AmpC de-repression (61/159, 38.4%), but of these only 9 relapsed. Furthermore, in only 3 of the relapsed cases did 3GC resistance develop during treatment (1.9% of the total cohort) (see Additional file 1: Table S1 for detail).

**Table 1:** Antibiotic susceptibility of *Enterobacter* species causing bacteraemia

Species	% Isolates (Number)	% Susceptibility (number tested)									
		CRO	GENT	TZP	TIM	CAZ	FEP	SXT	CIP	IMI	MER
*Enterobacter cloacae*	76.7	65.6	81.1	77.8	60.7	57.6	94.8	74.3	94.3	100	98.1
	(122)	(122)	(122)	(63)	(107)	(85)	(96)	(113)	(122)	(41)	(105)
*Enterobacter aerogenes*	19.5	74.2	100	76.9	78.6	88.2	100	100	96.8	100	100
	(31)	(31)	(31)	(13)	(28)	(17)	(27)	(29)	(31)	(8)	(29)
Other *Enterobacter* spp*.*	3.8	66.7	83.3	100	75	50	100	83.3	100	100	80
	(6)	(6)	(6)	(3)	(4)	(4)	(4)	(6)	(6)	(2)	(5)
All species	100	67.3	84.9	78.5	64.7	62.3	96.1	79.7	95	100	97.8
	(159)	(159)	(159)	(79^a^)	(139)	(106)	(127)	(148)	(159)	(51)	(139)

*CRO* ceftriaxone, *GENT* gentamicin, *TZP* piperacillin-tazobactam, *TIM* ticarcillin-clavulanate, *CAZ* ceftazidime, *FEP* cefepime, *CIP* ciprofloxacin, *MER* meropenem, *IMI* imipenem, *SXT* trimethoprim-sulphamethoxazole

^a^Limited number of isolates as not routinely tested in all labs until 2010

Empirical combination therapy was common (73/159, 45.9%), most frequently employing ampicillin plus gentamicin (10/73, 13.7%). Excluding patients treated with combination therapy, the most commonly prescribed agents for empirical treatment, with in vitro activity against *Enterobacter* spp., were aminoglycosides (31/86, 36%) or a broad-spectrum BLBLI (ticarcillin-clavulanate or piperacillin-tazobactam) (12/86, 13.9%) followed by carbapenems (11/86, 12.8%). For definitive treatment, a carbapenem was most commonly used (45/159, 28.3%), followed by a fluoroquinolone (39/159, 24.5%), BLBLIs (21/159, 13.2%) and aminoglycosides (11/159, 6.9%) (Table 2). The median duration of treatment was 10 days (range 0 to 328 days; IQR 2–40 days) and did not differ significantly between cases and controls (*p* = 0.13, Wilcoxon rank-sum test). The mean SAPSII score was 34.7 (range 6–89; SD 13.6), which corresponds to a risk of in-hospital mortality of approximately 16.2%.

**Table 2:** Frequency of bacteraemia relapse by definitive treatment category

Definitive treatment category	Relapse (cases)	Non-relapse (controls)	Total
Carbapenem	8 (25.8%)	37 (28.9%)	45 (28.3%)
Quinolones	4 (12.9%)	35 (27.3%)	39 (24.5%)
Other^a^	6 (19.4%)	21 (16.4%)	27 (17.0%)
Piperacillin-tazobactam/ticarcillin-clavulanate	6 (19.4%)	15 (11.7%)	21 (13.2%)
Aminoglycosides	3 (9.7%)	8 (6.2%)	11 (6.9%)
No antibiotic treatment	2 (6.5%)	2 (1.6%)	4 (2.5%)
Trimethoprim/sulphamethoxazole	1 (3.2%)	3 (2.3%)	4 (2.5%)
Cefepime	1 (3.2%)	2 (1.6%)	3 (1.9%)
Ampicillin/amoxicillin	0 (0.0%)	2 (1.6%)	2 (1.3%)
Cephazolin	0 (0.0%)	1 (0.8%)	1 (0.6%)
Ceftriaxone/ceftazidime	0 (0.0%)	1 (0.8%)	1 (0.6%)
Trimethoprim	0 (0.0%)	1 (0.8%)	1 (0.6%)
Total	31	128	159

^a^Includes combination therapy

Results for the univariate and multivariate logistic regression are summarised in Table 3. In the final model, the strongest predictors of relapsed or persistent bacteraemia were the presence of immunosuppression (OR 2.70; 95% CI 1.14-6.44 *p* = 0.02) or a line-associated source of bacteraemia (OR 3.87; 95% CI 1.56-9.60, *p* = 0.004), whereas choice of definitive antibiotic therapy did not show a significant effect on the univariate analysis.

**Table 3:** Univariate and multivariate logistic regression of the effects of clinical variables on relapsed *Enterobacter* bacteraemia

Variable		Cases (relapse) N = 31	Controls (non-relapse) N = 128	Univariate model			Multivariate model		
				OR	95% CI	*P*	OR	95% CI	*P*
Age, mean (SD)		52.0 (17.0)	60.5 (18.6)	0.98	0.95-1.00	0.02*			
Sex	Female	16 (51.6%)	49 (38.3%)	*female as reference*					
	Male	15 (48.4%)	79 (61.7%)	0.58	0.26-1.28	0.18			
Medical Service	Med/surg	10 (32.3%)	82 (64.1%)	*general medical / surgical as reference*					
	Haem/onc	15 (48.4%)	30 (23.4%)	4.1	1.66-10.11	0.002*			
	Renal	6 (19.4%)	16 (12.5%)	3.08	0.98-9.66	0.06			
Definitive therapy	Standard	23 (74.2%)	105 (82.0%)	*carbapenem, quinolone, cefepime, aminoglycoside or co-trimoxazole as reference*					
	BLBLI	6 (19.4%)	15 (11.7%)	1.83	0.64-5.21	0.26			
	Inappropriate	2 (6.5%)	8 (6.2%)	1.14	0.23-5.73	0.87			
Source	Non-line source	8 (25.8%)	80 (62.5%)	*non-line source as reference*					
	Line-associated	23 (74.2%)	48 (37.5%)	4.79	1.99-11.56	<0.001*	3.87	1.56-9.60	0.004*
Immune suppression	Absent	12 (38.7%)	83 (64.8%)	*no immunosuppression as reference*					
	Present	19 (61.3%)	45 (35.2%)	2.92	1.30-6.56	0.009*	2.70	1.14-6.44	0.02*
ICU admission	Non-ICU	25 (80.6%)	99 (78.6%)	*non-ICU admitted as reference*					
	ICU	6 (19.4%)	27 (21.4%)	0.88	0.33-2.36	0.80			
Acquisition status	Community	2 (6.5%)	29 (22.7%)	*community-associated infection as reference*					
	Healthcare	29 (93.5%)	99 (77.3%)	4.25	0.96-18.87	0.06			
Region	NSW	15 (48.4%)	73 (57.0%)	*New South Wales region as reference*					
	Queensland	16 (51.6%)	55 (43.0%)	1.42	0.64-3.11	0.39			
De-repressed AmpC phenotype	Absent	22 (71.0%)	76 (59.4%)	*no de-repressed AmpC as reference*					
	Present	9 (29.0%)	52 (40.6%)	0.60	0.26-1.40	0.24			
SAPS II score, mean (SD)		37.9 (15.9)	33.9 (12.9)	1.02	0.99-1.05	0.14			
* Enterobacter* species	Other *Enterobacter* spp*.*	6 (19.4%)	31 (24.2%)	*other Enterobacter spp. as reference*					
	*E. cloacae*	25 (80.6%)	97 (75.8%)	1.33	0.50-3.54	0.57			

*significant *p* < 0.05

## Discussion

In this case–control study, 3.4% of patients with *Enterobacter* bacteraemia were found to have relapsed by 28 days. The most striking finding is the effect of bacteraemia source on the risk of this outcome; 74% of all relapsed cases had a line-associated source for the bacteraemia, and this variable had the greatest effect size in the multivariate logistic regression model (OR 3.87). Although data were not available to reliably determine the precise date of line-removal in the majority of cases, we would hypothesise delayed line removal as a likely reason for this association.

Given the well-described association with 3GC treatment and AmpC-mediated relapse when used for *Enterobacter* infections, this study also aimed to assess the effect of antibiotic choice on the risk of relapse. When compared with alternative effective therapies (such as carbapenems, cefepime, quinolones or aminoglycosides) BLBLIs were not significantly associated with bacteraemia relapse when used as definitive therapy. However, BLBLIs were used relatively infrequently for definitive therapy. Overall antibiotic use was heterogeneous and often included combination therapy, meaning that effects for individual agents were diluted. We defined BLBLI therapy as including ticarcillin-clavulanate and piperacillin-tazobactam. However, these agents have some important differences with respect to AmpC-enzymes. Clavulanate is a potent inducer of AmpC and a poor inhibitor, whereas piperacillin and tazobactam are only weak inducers of AmpC [33].

Many laboratories suppress susceptibility results for AmpC-producers (such as *Enterobacter* spp.) in favour of carbapenems or quinolones due to concerns of selecting for AmpC de-repressed variants. According to the findings of this study, this may not be justified as a routine policy, especially in cases where source control has been addressed or in the absence of significant immunosuppression. If BLBLIs are used against susceptible *Enterobacter* isolates there does not appear to be any clear association with microbiological failure; and failure with the emergence of AmpC-mediated resistance appears to be rare. However, given the small number of patients treated with BLBLIs in this study, the possibility of a type II error exists, and this needs further investigation in larger cohorts. The proportion of patients treated with BLBLIs was lower than expected, which compromised the power of the study to address the effect of antibiotic choice on outcome.

In this current era of emerging carbapenem-resistance, it is critical that alternatives to carbapenems are sought for common infections. Infectious disease practitioners in our region most frequently recommend carbapenems for AmpC beta-lactamase producing Enterobacteriaceae [34]. As such, it may be a significant driver of carbapenem use. Our findings would suggest that using a BLBLI when susceptibility is demonstrated, especially in immunocompetent patients in whom adequate source control has been achieved, is not clearly associated with microbiological failure. Further work would help to delineate patients for whom carbapenems may still be a superior choice.

Not only was the risk of relapsed *Enterobacter* bacteraemia low (3.4%) in this study, but in only 3 cases did resistance to 3GCs (a marker for AmpC de-repression) emerge during treatment. This figure is lower than described in some previous studies [8, 9]. There may be several reasons for this observed difference. Firstly, clinicians are more aware of this clinical phenomenon and 3GCs are currently rarely used as definitive treatment for *Enterobacter* bacteraemia. In this study only 3 of 159 (1.9%) bacteraemia events received either ceftriaxone or ceftazidime as definitive therapy, and in 2 of these 3GCs were given in combination with another agent. This may also reflect local laboratory practice for withholding susceptibility reporting for 3GCs against *Enterobacter*. Secondly, low rates of relapse may also result from better recognition of source control and the availability of other effective antibiotics, especially carbapenems, cefepime and fluoroquinolones. Other variables that may have influenced the low rate of relapse in this study might include the clinical characteristics of our patient population as well as long-standing infection control and antimicrobial stewardship programs.

The overall risk of *Enterobacter* bacteraemia with a de-repressed AmpC phenotype in our region was also relatively low over the study period, with only 38.4% of isolates being resistant to 3GCs (either ceftriaxone or ceftazidime). Very few isolates showed resistance to carbapenems, although MIC breakpoints were lowered during the study period. Both current and older clinical breakpoints may fail to detect the presence of carbapenemase in Enterobacteriaceae [35, 36]. Our findings may not be generalizable to populations encountering higher rates of baseline 3GC resistance, or frequent ESBL / carbapenemase acquisition in *Enterobacter* species.

Limitations of the study are acknowledged. Although the bacteraemia database was collected prospectively as part of routine surveillance, clinical and microbiological data extraction for *Enterobacter* cases was retrospective. Although a laboratory servicing several hospitals was used to identify cases, these occurred within a limited area in Australia and may not represent the diversity of infections and patient populations found in other states and countries. Whether all repeat bacteraemia episodes were true relapsed events or new infections is also not known, as isolates were not available for molecular typing. Equally, the presence of co-existing ESBLs was not sought systematically for isolates demonstrating resistance to 3GCs; inferring Amp-C de-repression based on the antibiogram alone may be unreliable. This study only examined relapsed bacteraemia, since such an endpoint is easily defined. However, the risk of relapsed infection and emergent resistance from other clinical sites infected with *Enterobacter* may be different and care should be taken when extrapolating from bacteraemia data alone.

Given the inherent limitations of retrospective studies, it is hoped that the efficacy of BLBLIs or other carbapenem-sparing options for *Enterobacter* infections could be addressed in large prospective observational studies or even randomised controlled trials (RCTs). A pilot RCT comparing piperacillin-tazobactam with meropenem for bloodstream infections caused AmpC-producers is currently recruiting (MERINO-2 Trial; ClinicalTrials.gov identifier: NCT02437045).

## Conclusions

In a cohort of patients with bacteraemia caused by *Enterobacter* species, the overall risk of relapsed bacteraemia was low. Patient factors, such as a line-source for the bacteraemia or the presence of immunosuppression, were the strongest predictors of relapse. Use of a BLBLI agent as definitive therapy was not associated with treatment failure, but the total number of patients treated with a BLBLI was relatively low. Although this would suggest that susceptibility in vitro should translate into in vivo efficacy for BLBLI agents, especially if the patient has adequate source control, further studies are warranted to determine if BLBLIs are a safe and effective carbapenem-sparing option, especially for patients with severe disease, a complex focus of infection or immune compromise. Our data also reinforce the importance of line removal when suspected as the source for bacteraemia, especially for isolates with the genetic capacity to respond rapidly to antimicrobial exposure.

## Additional file

Additional file 1: Table S1. Clinical details of relapsed Enterobacter bacteraemia cases with emergence of antibiotic resistance. (DOCX 17 kb)

## Abbreviations

3GC: Third generation cephalosporin
BLBLI: Beta-lactam/beta-lactamase inhibitor
CLSI: Clinical and laboratory standards institute
ESBL: Extended-spectrum beta-lactamase
EUCAST: The European committee on antimicrobial susceptibility testing
ICU: Intensive care unit
MALDI-TOF: Matrix assisted laser desorption/ionization time of flight
MDR: Multi-drug resistant
MIC: Mimimum inhibitory concentration
OR: Odds ratio
RCT: Randomised controlled trial
