# Supplementary material for: Risk factors for relapse or persistence of bacteraemia caused by Enterobacter spp.: a case–control study
Source: Antimicrob Resist Infect Control. 2017 Jan 21;6:14. doi: 10.1186/s13756-017-0177-0 (PMC5251334; doi:10.1186/s13756-017-0177-0)
Supplement: Additional file 1: Table S1. — Clinical details of relapsed Enterobacter bacteraemia cases with emergence of antibiotic resistance. (DOCX 17 kb) [file 13756_2017_177_MOESM1_ESM.docx]

**Risk factors for relapse or persistence of bacteraemia caused by *Enterobacter* spp.: a case-control study**

**Supplementary Table 1:** Clinical details of relapsed *Enterobacter* bacteraemia cases with emergence of antibiotic resistance.

| Hospital | Age / sex | Source | Co-morbidity | Empirical therapy | Definitive therapy | Days to relapse post initial +ve BC | Emergent antibiotic resistance |
| --- | --- | --- | --- | --- | --- | --- | --- |
| Mater (NSW) | 45 F | Unknown | Leukaemia (AML) | Ceftazidime + Ciprofloxacin | Ceftazidime + Ciprofloxacin | 9 | CRO S → R  TIM S → R |
| Hunter (NSW) | 60 M | Line-associated (Dialysis) | Renal dialysis | Unknown | Gentamicin | 5 | TIM S → R* |
| Hunter (NSW) | 43 M | Line-associated (CVL) | Trauma | Ticarcillin-clavulanate + Gentamicin | Meropenem | 4 | CRO S → R  CAZ S → R  TIM S → R  GEN S → R  SXT S → R |
| Mater (NSW) | 78 M | Unknown | Malignancy | Ticarcillin-clavulanate + Gentamicin | Ticarcillin-clavulanate | 4 | TIM S → R* |
| RWBH (QLD) | 78 M | Line-associated | Renal dialysis | Gentamicin | Meropenem | 20 | CRO S → R  TIM S → R |
| RBWH (QLD) | 45 M | Line-associated (Port) | Leukaemia (ALL) | Piperacillin-tazobactam + Tobramycin | Piperacillin-tazobactam + Tobramycin | 7 | AK S → R |
| RBWH (QLD) | 44 F | Line-associated (Hickman) | Leukaemia | Piperacillin-tazobactam | Meropenem | 21 | CIP S → R |

Abbreviations: NSW = New South Wales, QLD = Queensland, F = Female, M = male, CVL = central venous line (temporary), AML = Acute myeloid leukaemia, ALL = Acute lymphoblastic leukaemia, CRO = ceftriaxone, TIM = Ticarcillin-clavulanate, CAZ = ceftazidime, GEN = gentamicin, SXT = trimethoprim-sulphamethoxazole, AK = amikacin, CIP = ciprofloxacin

*note, in both cases CRO remained susceptible
